# Supplementary material for: Impact of communication anxiety on L2 WTC of middle school students: Mediating effects of growth language mindset and language learning motivation
Source: PLoS One. 2025 Jan 14;20(1):e0304750. doi: 10.1371/journal.pone.0304750 (PMC11731871; doi:10.1371/journal.pone.0304750)
Supplement: S1 File — (DOCX) [file pone.0304750.s001.docx]

**Items for communication anxiety (CA)**

CA1: It embarrasses me to volunteer answers in my language class.

CA2: I tremble when I know that I am going to be called on in English class.

CA3: The more I study for English test, the more confused I get.

CA4: I keep thinking that the other students are better at English than I am.

CA5: I felt very self-conscious about speaking English in front of other students.

CA6: English oral class moves so quickly that I worry about getting left behind.

CA7: I feel more tense and nervous in my English speaking class than in my other classes.

CA8: I feel overwhelmed by the number of mistakes I made in English oral communication.

CA9: I am afraid that the other students will laugh at me when I speak English.

**Items for growth language mindset (GLM)**

GLM1: I can always substantially change my English language ability.

GLM2: If I work hard at English, I will always get better results.

GLM3: I can always change your English language ability as long as I work hard at it.

GLM4: Everyone could do well in English if they try hard, whether they are young or old.

**Items for language learning motivation (LLM)**

LLM1: I think that I am doing my best to learn English.

LLM2: I usually spend lots of time studying English.

LLM3: I can overcome the difficulties and remove interferences when I learn English.

LLM4: The things I want to do in the future require me to use English.

LLM5: Whether my teacher is strict with me or not, I will study English consciously.

LLM6: Compared to most of my classmates, I think I study English relatively hard.

LLM7: I have my own after-class English learning plan.

**Items for willingness to communicate (WTC)**

WTC1: If I encountered non-native speakers of English (Japanese, Korean,French, etc.) ,I would talk to them in English.

WTC2: To practice my English, I am willing to talk in English with my English teacher in the class.

WTC3: I am willing to ask questions in English in the English classes.

WTC4: I am willing to talk and express my opinions in English in the class when all my classmates are listening to me.

WTC5: I am willing to have pair and group activities in the class so that I can talk in English with my classmates.
