# Supplementary material for: Impact of communication anxiety on L2 WTC of middle school students: Mediating effects of growth language mindset and language learning motivation
Source: PLoS One. 2025 Jan 14;20(1):e0304750. doi: 10.1371/journal.pone.0304750 (PMC11731871; doi:10.1371/journal.pone.0304750)
Supplement: Attachment — Submitted filename: Response to Reviewers.docx [file pone.0304750.s003.docx]

Manuscript ID: PONE-D-24-19380

Dear editor and reviewers:

We would like to begin by expressing my sincere gratitude for the thorough and insightful review of our manuscript titled “Impact of communication anxiety on L2 WTC of middle school students: Mediating effects of growth language mindset and language learning motivation” (Manuscript ID: PONE-D-24-19380). We appreciate the time and effort taken by both you and the reviewers to provide detailed feedback and constructive criticism. The comments and suggestions offered have been invaluable in highlighting areas that require our attention and have provided us with a clear direction for revising and improving our work. We have studied comments carefully and have made corrections. Below we indicate that how we responded to each of your comments.

The questions proposed by the reviewers were in bold in black, and our responses to the questions were in red.

**Response to the editor**

**Please include your full ethics statement in the ‘Methods’ section of your manuscript file. In your statement, please include the full name of the IRB or ethics committee who approved or waived your study, as well as whether or not you obtained informed written or verbal consent.**

Response: Thank you for informing us. We have included this part to **Section 3.3 Data collection**. Please see the **Mark-up version** from line 358 to line 366.

**Response to Comments of Reviewer 1**

**Major points**
**1. Page 31, lines 627-633: In these lines, it is mentioned that the current study found that anxiety indirectly affects WTC in L2 through student motivation, which contrasts with the paths identified by Khajavy et al. (2016), who hypothesized a bidirectional role of anxiety with motivation. Perhaps adding a few lines explaining more clearly why this bidirectionality was hypothesized could further strengthen your results.**

Response：

We extend our sincere gratitude for the reviewer’s valuable insights, which are greatly acknowledged. Below please find the detailed elaboration from line 759 to line 765 in the **Mark-up Version**: “However, our finding of communication anxiety influencing L2 WTC indirectly through learner motivation different from the pathways identified by Khajavy et al. [62] and Yu [63], where L2 motivation influenced L2 WTC via L2 anxiety. These findings do not conflict but rather reinforce the bidirectional nature of the relationship between negative emotions and motivation. Moreover, learner affect may serve as a predictor and/or mediator in various communication models”.

**2.** **Page 23-24, lines 467, 485-488: Nine hypotheses and three indirect paths are mentioned. However, according to the introduction, the number of hypotheses should be 7 and the number of hypothesized indirect paths should be 2 (also looking at Table 3). Hence, there is an inconsistency that deserves to be corrected or explained.**

Response:

We concur with the accuracy of your suggestions, and have duly incorporated them. The manuscript has been revised in line with your guidance, with changes clearly annotated. We wish to reiterate our appreciation for your contributions. For details, please see line 579 to 581 in the **Mark-up Version**.

**Minor points**

Response:

Upon careful consideration of the thorough and insightful comments offered by the reviewer, we have implemented the subsequent revisions to address the noted issues within our manuscript. Specifically, we have rectified typographical errors pertaining to punctuation, and spacing, in addition to addressing grammatical inaccuracies. We express our sincere gratitude for the meticulous guidance provided.

**Response to Comments of Reviewer 2**

**Major points**

1. **Research ethics statement**

Response:

We extend our apologies for not including the ethical review report for the project in our previous submission. Due to the necessity for our institutional ethics review board to retain the original copy of the ethical review report for archival purposes, we had not previously made a duplicate of the original ethical review approval form. In compliance with your request, I have now added the copy. It is now furnished as supporting material to the submitted material. We appreciate your valuable suggestions.

**2.Data availability——the authors have stated that the data is available in the manuscript and supporting documents, but a link to where the data set is stored has not been provided. It may be that the authors have not understood the requirement to provide a minimal data set behind the results reported in the manuscript.**

Response:

Thank you so much for your advice. During the online submission process, this component was inadvertently omitted from the initial submission. We deeply regret any inconvenience this may have caused and appreciate your understanding. Now the dataset in question has been uploaded as supporting material.

**2.Writing – the manuscript would benefit from copyediting as there were grammatical errors throughout the manuscript and sentences / sections that could benefit from better organisation.**

Response:

We extend our sincere gratitude for the reviewer's diligent and thoughtful guidance pertaining to linguistic and mechanical aspects of our manuscript. Following the meticulous examples offered by the reviewer, we have undertaken a thorough revision of each item, ensuring a comprehensive enhancement of the text. Introduction

**Grammatical errors**

**40-43;60;66;67;124;133-136;148;164;200-204;208**

Response:

The above-mentioned grammatical errors have been corrected.

**Word choice**

**50-51**

Response:

According to the reviewer’s advice, we use the down toner “may” to soft the tone. Please see line 43 in the Mark-up version.

**74-77**

Response:

We concur with the reviewer's perspective. We intend to emphasize the examinations that focus on receptive skills, encompassing a broad range of passages designed to assess learners' abilities in listening and reading comprehension. “Both instructors and students tend to prioritize receptive skills, such as listening comprehension, reading comprehension, over writing and speaking.” For details, please see line 72 to 73 in the Mark-up version.

**Content**

**Line 83-92;109;165;211**

Response:

We express our gratitude to the reviewer for the thorough and insightful feedback. We have made the necessary subsequent amendments in accordance with the reviewer's recommendations. For details, please kindly turn to the Mark-up version

**Line 83-92 shortened version**

Response:

We shortened this part into the following sentences (For details, please see Mark-up version line 81 to line 92). “It is expected to provide substantial theoretical and practical implications for augmenting L2 WTC of middle school students. Additionally, it is also expected to highlight the role of learner-related factors in cultivating L2 WTC, offering practical insights for the development of educational strategies aimed at enhancing L2 WTC

**Line 109**

Response:

Thank you for the constructive advice. We added further elaboration on ideal L2 Selves in the Mark-up version. Please see line 109 to line 111. “The research demonstrated that ideal L2 selves, which encompassing learners' aspirations and their envisioned future linguistic identities significantly correlates with WTC in English among Japanese high school students, both in and out of the classroom.”

## Methods

**Line 285;267;287;289;293;298291;296;281-283;299-301**

Response:

Grammatical errors as well as technical problems from the above-mentioned lines have been corrected and solved according to the reviewer’s advice.

**Line 271-301**

Response:

Detailed modification to the validated scales were added from line 312 to line 319 in the mark-up version: “(1) Items were rephrased to reflect cultural norms and experiences specific to middle school students;(2) Language were simplified to ensure it is accessible to youngsters; (3) The number of items were reduced as well as structure simplified to reduce the cognitive load. The English questionnaire was translated into Chinese by the first writer and the translated questionnaire items were subsequently submitted to three linguistic experts for validation to ensure their accuracy. Based on the feedback provided by the experts, the language of the translated items underwent further refinement.”

## Results

**Line 313;366;381;415;**

Response:

Thank you so much for pointing out. The issues pertaining to spacing and language have been meticulously revised based on the reviewer's constructive feedback.

**Line 414-419**

Response:

Detailed fit indices were added in the Mark-up version from line 499 to line 505. The following are the added information:

“For CFI and TLI, a value of 0.90 or above is generally considered to indicate a satisfactory fit, while a value of 0.95 or above suggests an excellent fit. For RMSEA, a value of 0.08 or less is typically considered to indicate a satisfactory fit, and a value of 0.06 or less suggests an excellent fit. Similarly, for SRMR, a value of 0.08 or less is generally considered to indicate a satisfactory fit, and a value of 0.06 or less suggests an excellent fit [52].”

**Line 485-488**

Response:

We have meticulously reviewed the hypotheses and implemented the necessary adjustments as per the reviewer's recommendations. For details, please see the Mark-up version from line 579 to line 581.

## Discussion

**Line 499;518;542;554;571;587;607**

Response

Subheadings were added respectively. Please kindly turn to line 593 to line 594;line 627 to line 629;line 664 to line 666; line 688 to line 690;line 707 to line 709;line 726 to line 727, and line 751 to line 754.

**Line 502;519;520;523-525;547;604;605;650**

Response:

First and foremost, we express our sincere gratitude for the patient and meticulous suggestions provided by the reviewer. We found the feedback to be highly constructive and have accordingly made the necessary revisions.

**Line 533-541**

Response:

We thank the reviewer for the advice. We added the following line in the Mark-up version (Line 654 to line 662). “which can significantly influence their engagement in communicative activities. Therefore, as Lou & Li [58] have posited that growth mindset may not yield the desired outcomes or may even have negative consequences in societies where fixed-mindset norms are prevalent. Some cultures may place a higher value on humility and effort rather than on inherent talent or potential for growth. Additionally, a growth mindset may not necessarily enhance WTC in learning contexts that are highly competitive or discourage risk-taking. Thus, under the above-mentioned circumstances, a growth mindset might not translate into increased WTC.”

**Line 568;581,666**

Response:

We appreciate your valuable feedback. Upon careful consideration, we think that it might be more beneficial to include suggestions for teachers and educators within the section of “**Theoretical and pedagogical implications**”. Therefore, we have decided to incorporate the “Advice for teachers and educators” within Section 5.8. Please kindly turn to the Mark-up version from line 782 to line 812.

we included actionable strategies that educators can employ to mitigate communication anxiety and foster greater language learning motivation among their students.

**Line 678-683**

Response:

Thank you for the advice. The role of technology in enhancing student engagement and boosting language learning motivation is supported by specific literature. Please kindly turn to line 807 to line 812 in the Mark-up version. “Educators are recommended to adopt various measures to boost learners’ motivation, such as digital storytelling [64]; digital games [65]; online cooperative learning [66]. In the digital era, innovative educational technologies, including artificial intelligence (AI), augmented reality (AR), and virtual reality (VR) can be to leveraged to enhance students’ engagement, and strengthen their external and internal motivation [67,68], thereby advancing their proficiency in L2 and enabling learners to attain their language learning objectives.”

Once again, we thank both the reviewers and the editor for your helpful comments. We hope that our responses and the revised version of the paper are satisfactory and address your concerns adequately.

Please feel free to contact us if you have any further questions.
